# Effect of the Addition of Steel Fibers on the Bonding Interface and Tensile Properties of Explosion-Welded 2A12 Aluminum Alloy and SS-304 Steel

**DOI:** 10.3390/ma16010116

**Published:** 2022-12-22

**Authors:** Yao Chen, Yonghong Gao, Chuanxiang Guo, Yanping Guo, Zhijun Guo, Yingbin Liu, Tiansheng Liu

**Affiliations:** 1 School of Environment and Safety Engineering, North University of China, Taiyuan 030051, China; 2 Department of Environmental and Safety Engineering, Taiyuan Institute of Technology, Taiyuan 030008, China; 3 Shanxi Fenxi Heavy Industry Co., Ltd., Taiyuan 030027, China

**Keywords:** steel fiber, explosive welding, numerical simulation, bonding interface

## Abstract

First of all, the explosion-welding method was adopted to prepare steel fiber-reinforced steel-aluminum composite plates. Secondly, the smooth particle hydrodynamic (SPH) method was used to investigate the effect of introducing steel fibers to a vortex region created at the bonding interface of the steel-aluminum composite plate. Thirdly, the following conclusions were drawn through an analysis of the vortex region with the assistance of scanning electron microscopy and energy-dispersive X-ray spectroscopy. A brittle intermetallic compound FeAl was produced in the vortex region in an environment characterized by high temperature, high pressure, and high strain rate, resulting in cracks, holes and pores. In addition, the hardness of the vortex area was less than the estimated value, which is mainly because the main element in the vortex area was 2A12 aluminum with low hardness, and there were cracks, holes, pores and other defects that caused hardness reduction. Although the addition of steel fibers caused defects at the bond interface, the addition of steel fibers was effective in improving the tensile resistance performance of steel-aluminum composite panels to a certain extent. In addition, the larger the fiber diameter, the more significant the increase in tensile resistance.

## 1. Introduction

With the rapid development of industry, more and more regions need to produce special equipment by machining high-quality metals into sheets with excellent anticorrosion, antioxidation, and mechanical properties. If all these devices are made of high-quality metals, their costs will be inevitably high, resulting in unnecessary waste. Therefore, replacing high-quality metals with bimetallic materials is the most ideal solution. SS-304 stainless steel is characterized by high strength, impact resistance, reliable performance, and good welding and riveting performance, but it has a lot of shortcomings, such as high maintenance cost, heaviness, and large production energy consumption. However, 2A12 aluminum material has a lot of advantages, including lightness, good electrical conductivity, ease of extension, corrosion resistance, and few toxic and side effects. Bimetallic materials have the advantages of two metal components, so the application of steel-aluminum composite plates can reduce the mass of SS-304 stainless steel and improve its corrosion resistance. Explosive welding is a solid-phase welding method, which can be used for the welding of double-layer or multilayer composite plates of the same or different metals [1,2,3,4,5,6]. Compared with traditional diffusion welding [7,8], light welding [9,10], magnetic pulse welding [11,12], and hot rolling welding [13,14], the explosive welding method still has very good welding quality in the case of large differences in the points or mechanical properties of the welded metal, so it is extensively used in metal welding. Because of its high tensile strength, low price, and outstanding corrosion resistance, steel fiber is widely used to reinforce concrete and improve its protective performance. In this paper, explosive welding was used to produce a new fiber-reinforced steel aluminum composite plate by combining the advantages of SS-304 stainless steel, 2A12 aluminum, and steel fiber.

Extensive studies have been conducted on steel-aluminum composite plates, with outstanding achievements. In 1983, Kotov V A studied [15] unidirectional fiber-reinforced composites composed of steel wires based on Amr6 aluminum alloy, and the results revealed that under uniaxial or biaxial loads, the strength of the fiber-reinforced composites was mostly dependent on the bonding strength of the wire and the base plate, as well as the internal geometric installation, and when the load was along the wire, the effect was relatively obvious. Therefore, fiber-reinforced composites and tubular products prepared by explosive welding have sufficient strength under various loads. Zhou et al. [16] successfully prepared steel fiber-reinforced composite plates and used numerical simulation and experimental methods to study the impact of the addition of steel fiber on the antipenetration performance of the composite plate. It was concluded that the addition of the fiber-reinforced phase was improved the antipenetration performance of the composite target plate, and the reduced fiber distribution spacing and orthogonal arrangement distribution were helpful in improving the antipenetration performance of the target plate. Wang et al. [17] applied numerical simulation and experimental methods to study the impact of carbon fiber-reinforced polymer laminates under high-speed impact. The results showed that within a certain impact velocity range, carbon fiber composite laminates had the advantage of replacing metal plates to resist high-speed impact. Mahfuz et al. [18] comprehensively studied the antipenetration performance of multilayer ceramic–rubber–glass fiber composite target plates against high-velocity projectiles by numerical simulation and hydrogen gun experiments, and discussed the failure mode of the target plate at ballistic limit velocity. The addition of fiber can significantly increase the difficulty of explosive welding, so the experiment needed to calculate the dynamic parameters of explosive welding in advance to guarantee the rationality of the parameters.

The effects of steel fiber with different diameters on the interface and mechanical properties of steel-aluminum composite sheets were studied. In the early stage, the SPH method in Autodyn software was used to study the influence of high-temperature and high-pressure environments on steel fiber and composite interface to simulate the explosive welding experiment. Later, scanning electron microscopy and energy-dispersive spectroscopy were adopted to systematically study the changes in the bonding interface and the formation of intermetallic compounds after adding steel fibers. The effect of the addition of steel fiber on the hardness of the base plate was further studied by microhardness tests. Finally, a universal testing machine was used to verify the impact of the addition of steel fibers on the tensile properties of steel-aluminum composite plates.

## 2. Materials and Methods

**Figure 1:**
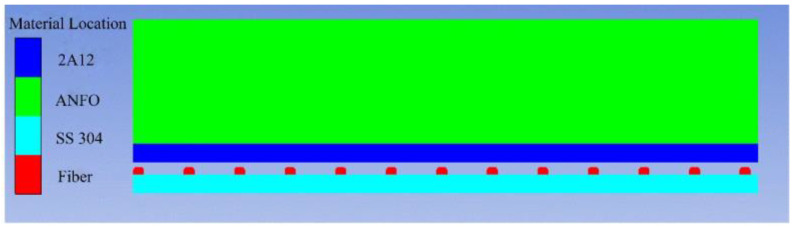
Explosive welding model.

Ansys/Autodyn software was used to carry out simulation experiments on SS-304 and 2A12 aluminum composite plates prepared by explosive welding. The SPH method can extensively simulate large deformation problems such as disintegration, fragmentation, solid spalling, and brittle fracture of continuum structures and avoid algorithm coupling, so it is very suitable for numerical simulation of explosive welding of multilayer metal plates [19,20]. Therefore, the SPH method can simulate the interaction of jet particles in the preparation of steel fiber-reinforced steel-aluminum composite plates by explosive welding and provide reasonable and effective experimental parameters for the following explosive welding experiment. As the contrast between the plate thickness and length width was large, a two-dimensional plane model was constructed. To save the calculation time of the model, the length of the base plate, aluminum composite plate, and explosive was all set to 40 mm. The particle size was set to 10 μm, and a total of 1.1815 million particles were generated to obtain accurate numerical results. The specific model is shown in Figure 1, where the density of the ANFO explosive is 0.6821 g/cm^3^, the explosive height is 20 mm, and the detonation velocity is 2430 m/s. The specific parameters of ANFO are shown in Table 1.

### Experimental Procedure

**Figure 2:**
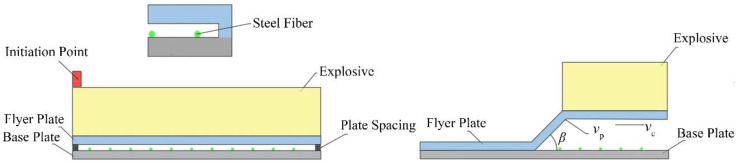
Welding diagram.

Explosive welding mainly includes: directly in the atmosphere, in water, and in rough vacuum. Compared with explosive welding experiments in low vacuum and water, explosive welding experiments in the air have a simpler assembly process, use lower detonation velocity of explosives, and are safer and more efficient. Therefore, in this paper, explosive welding experiments in the air [21,22,23] were selected. As shown in Figure 2, taking the SS-304 stainless steel with a size of 200 mm × 300 mm × 1 mm as the base plate, and the 2A12 aluminum plate with a size of 200 mm × 300 mm × 1 mm as the flyer plate, a steel fiber was evenly wound on the base plate every 5 mm to ensure that the length of the steel fiber was the same as that of the base plate, and the standoff distance between the base plate and cladding plate was 4 mm. Then, the base plate, flyer plate, and steel fiber were welded together by explosive welding. The performance parameters of steel and aluminum are shown in Table 2. S20910 is the material parameter of steel fiber and SS 304 is the material parameter of the base plate. In this experiment, the diameter of the steel fibers needs to be strictly controlled and the fibers are exposed to extrusion and extreme thermomechanical conditions in the welding process. The mechanical properties of the fibers therefore need to be good and the hot drawing process produces steel fibers of a more uniform size and high precision. Because the working temperature is above the recrystallization temperature, the resulting process hardening phenomenon is eliminated by recrystallization, the internal stress of the steel fibers is also eliminated, the toughness and plasticity will be better, so that the welded composite plate can better reflect the impact of the fibers on the composite plate. While cold rolled out of the steel plate as the base plate of the explosion welding, it ensures the strength and hardness of the base plate, in line with the experimental design. SS 304 steel as the base plate ensures the strength and hardness of the base plate, in line with the experimental design. The abovementioned metal materials were purchased by the teachers of the research group in Shenzhen Hongwang Mold Co., Ltd., Guangzhou, China.

Explosive welding aims to connect weldments by using the impact force generated by the explosion to cause the rapid collision of the weldments. The three most critical parameters are the impact velocity of the composite plate *v*_p_, the collision angle *β*, and the moving speed of the collision point *v*_c_. The moving speed of the collision point is equal to the detonation velocity of the explosive. Using 2# rock ammonium nitrate explosive, the parallel explosive welding experiment was used, satisfying the following relationship [26]:

(1) $$R=\frac{\rho_{e}h_{e}}{\rho_{f}h_{f}}$$

(2) $$R=\frac{C}{m}$$

(3) $$M=C\cdot S_{e}$$

(4) $$v_{p}=\sqrt{2E}{\left[\frac{{\left(1+2/R\right)}^{3}+1}{6\left(1+1/R\right)}+\frac{1}{R}\right]}^{-\frac{1}{2}}$$

(5) $$\sqrt{2E}=\frac{v_{d}}{3.08}$$

(6) $$v_{p}=2v_{d}\sin\frac{\beta }{2}$$

where *v*_p_ is the impact velocity; 2*E* is the Gurney energy; m is the mass of the flyer plate; *R* is the explosion ratio; *ρ*_e_ is the density of the explosive; *ρ*_f_ is the density of the flyer plate; *h*_e_ is the height of the explosive; *h*_f_ is the thickness of the flyer plate; *C* is the explosive mass per unit area; *S*_e_ is the explosive area, which is the same as the surface area of the flyer plate; *M* is the explosive payload; *v*_d_ = *v*_c_ equals the detonation velocity of the explosive. Therefore, the detonation velocity is 2400 m/s [27]. *v*_p_ = 668 m/s, *β* = 17.5°.

## 3. Results and Discussion

### 3.1. Interface Evolution Mechanism

The bonding of explosive welding is divided into three categories: (1) direct bonding of metals; (2) forming a uniform and continuous melting layer; and (3) undulate bonding, which is the most common form. In undulate bonding, because the molten material at the bonding interface is retained in the vortex and is periodically and discontinuously separated, when there is an external load, the microcrack source generated by the interface defects in the melting tank is not easily propagated. Therefore, the most ideal method is tiny undulate bonding. Many experiments and studies have shown that to realize high-quality explosive welding, the following three requirements should be met: (1) undulate bonding can be obtained under certain collision conditions, that is, the impact velocity *v*_p_ and the impact angle *β* meet the explosive welding window; (2) there must be jet formation when welding, so that the interface can be self-cleaning, exposing the fresh surface; and (3) a fine and uniform corrugated interface or a flat interface with sufficient strength is formed. Finally, to better understand this problem, SPH simulation was introduced to study the detailed evolution process of the steel-aluminum interface.

**Figure 3:**
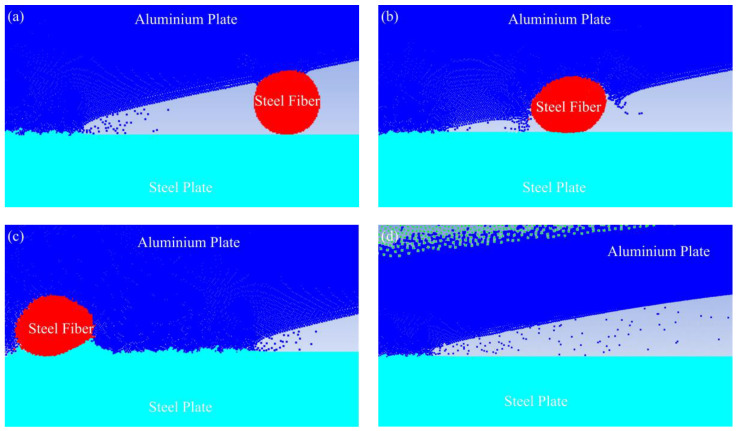
Evolution mechanism of welding interface: (**a**) time 3.5 μs; (**b**) time 4.0 μs; (**c**) time 4.5 μs; (**d**) time 4.0 μs.

**Figure 4:**
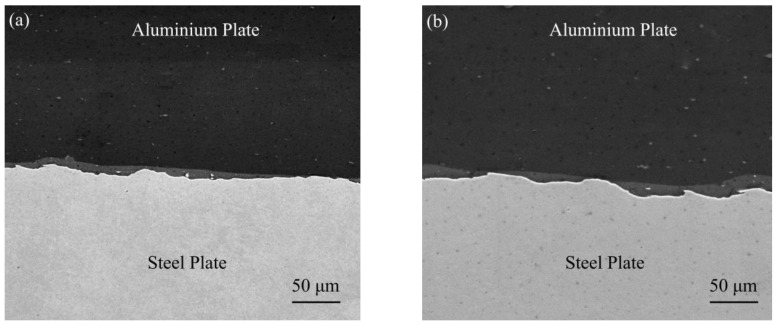
SEM images of explosion welded interfaces: (**a**) for steel fiber diameter 0.5 mm; (**b**) without steel fiber.

Figure 3a–c shows the evolution mechanism of the influence of steel fibers on jet particles. As shown in Figure 3a, during the welding process, the incident jet consisted of two layers on the surface. These particles ejected from the flyer plate moved obliquely downwards and acted on the base plate surface, thereby compressing the base plate, causing it to form a depression, forming a corresponding bulge on the base plate surface, and generating a forward jet. The incident jet could also remove oxides and other impurities on the surface, and help to establish ideal welding conditions under the circumstance of original clean contact [28]. However, after the detonation of the explosive, the explosive product formed a high-voltage pulse load, which directly acted on the flyer plate. Then, the flyer plate accelerated and reached a speed of several hundred meters/second in a few microseconds, starting from the starting end, collided with the base plate in turn, and formed a certain angle. At this time, the fixed included angle formed between the flyer plate, the steel fiber, and the base plate prevented the jet from continuing to act on the surface of the base plate, so that a large number of particles converged on the side of the steel fiber, as shown in Figure 3b. As the steel fiber spacing was 5 mm, when the jet was blocked, the new jet continued to generate. According to Figure 3c, the process of the jet from being blocked to being regenerated was repeated. Figure 3d is a schematic diagram for simulating jet particles without steel fibers. By comparing with Figure 3a,c, both the number of jet particles and the distance of propagation in Figure 3d were much larger than those in Figure 3a,c. It should be emphasized that Yang et al. [29] studied the evolution mechanism of the Ag–Fe welding interface by SPH simulation. The results showed that the movement direction of these jet particles was the main reason for the formation of the undulating interface, which explained why the undulating structure formed at the interface of the steel-aluminum composite plate with a steel fiber diameter of 0.5 mm in Figure 4a was more uniform than that without steel fibers in Figure 4b, and the wavelength and wave height in Figure 4b were greater than those in Figure 4a.

**Figure 5:**
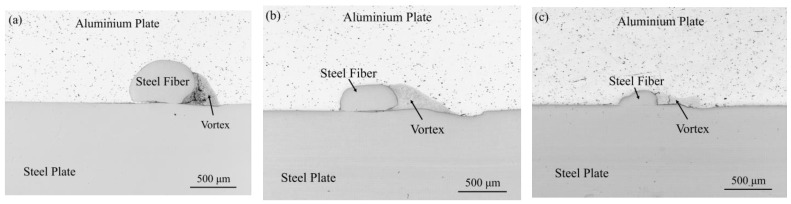
Schematic cross-section of steel fibers with different diameters: (**a**) steel fiber diameter: 0.5 mm; (**b**) steel fiber diameter: 0.35 mm; (**c**) steel fiber diameter: 0.25 mm.

**Figure 6:**
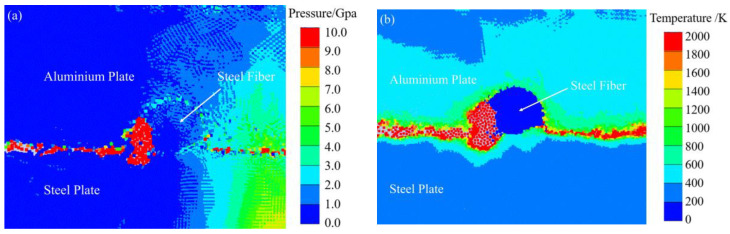

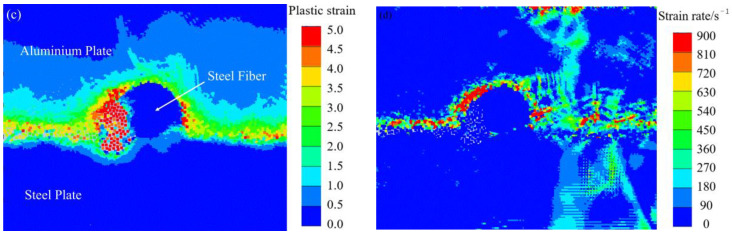
Thermodynamic state of bonding interface during explosive welding: (**a**) pressure; (**b**) temperature; (**c**) plastic strain; (**d**) strain rate.

**Figure 7:**
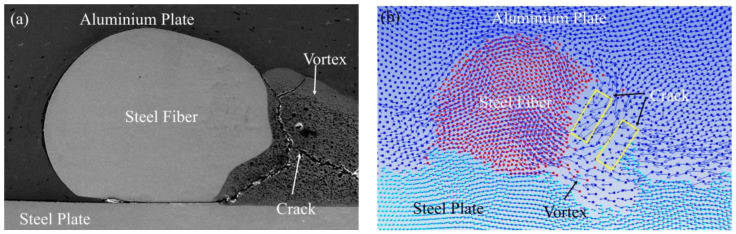
SEM and simulation results of vortex region: (**a**) SEM of steel fiber with a diameter of 0.5 mm; (**b**) numerical simulation of steel fiber diameter of 0.5 mm.

The thermodynamic state of the explosive welding process was simulated by the SPH method to better understand the interface evolution. Figure 5 shows the cross-sections of steel fibers with diameters of 0.5 mm, 0.35 mm, and 0.25 mm under an optical microscope. In the figure, three groups of steel fibers all showed a deformation to varying degrees, but all maintained a complete fiber structure. Among them, the addition of steel fibers with a diameter of 0.5 mm produced the most obvious vortex area and cracks, so it was taken as an example to analyze the impact of the addition of steel fibers on the bonding interface of the composite plate by combining with numerical simulation. Figure 6 is a schematic diagram of the thermodynamic state during the explosive welding process. As shown in Figure 6a, when the jet particles filled the unilateral side of the steel fiber, a high-temperature and high-pressure environment was formed, with a pressure of up to 10 GPa. For the three materials, the pressure was much greater than the yield strength of 235 MPa, so the steel fiber directly contacting the jet particles would behave as a fluid and undergo a strong plastic deformation. Figure 6b shows the temperature field. A high-temperature region was constituted by the molten jet particles on the steel fiber side, and combined with the uneven thin melt formed by the molten jet particles at the interface, a lot of heat accumulated in these two areas because time was not enough to spread heat. Figure 6c shows the maximum plastic deformation on one side of the steel fiber, where the maximum deformation occurred on the steel fiber side blocking the jet advance, with a maximum strain rate of 5. Figure 6d shows that the ultrahigh strain rate occurred at the steel fiber surface and the welding interface was up to 900/s. To sum up, the explosive welding process occurred in an extreme environment with high temperature, high pressure, large strain, and superhigh strain rate, and this extreme thermodynamic state was closely related to the bonding interface. These high-temperature molten particles driven by the kinetic energy of the composite plate were correlated with the combined effects of the two materials, including plastic deformation, friction, shear, and stirring [30,31]. Since the time was not enough to reduce the large-scale heat, these molten particles could not be rapidly solidified. Then, the residual velocity forced the molten particles on one side of the steel fiber to stir and mix strongly, and finally a vortex region with cracks was formed, as shown in the yellow region in Figure 7b. Yang, Zhang, Bazarnik et al. [32,33,34] proved that the formation of the vortex structure might be due to the good ductility and high density of steel. Figure 7a shows the cross-section of the steel fiber-reinforced steel-aluminum composite plate with a steel fiber diameter of 0.5 mm. For the shape and cracks in the vortex region in the figure, by comparing it to Figure 7b, it was found that the numerical simulation results were in good agreement with the experimental observations.

### 3.2. Energy Spectrum Analysis

**Figure 8:**
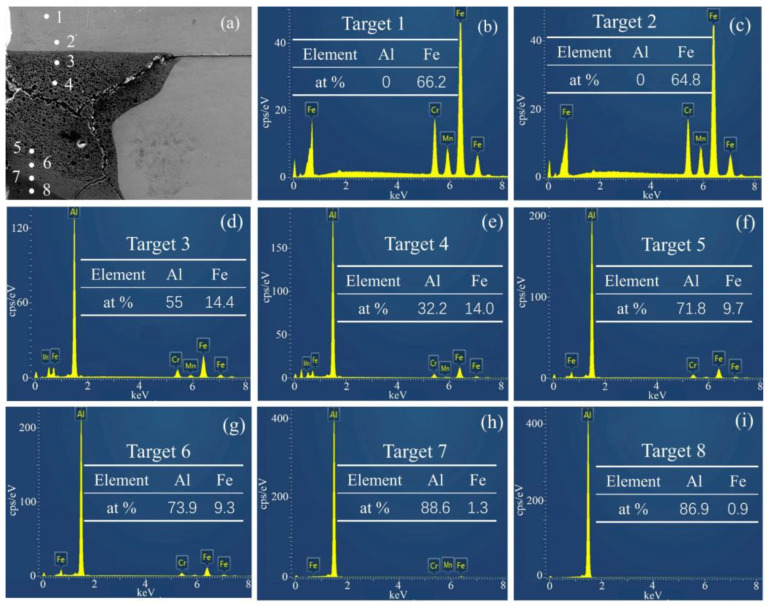
Position points and numerical results of welding interface analyzed by EDS in SEM image: (**a**) Schematic diagram of SEM positions of 8 points; (**b**) Scan result of point 1; (**c**) Scan result of point 2; (**d**) Scan result of point 3; (**e**) Scan result of point 4; (**f**) Scan result of point 5; (**g**) Scan result of point 6; (**h**) Scan result of point 7; (**i**) Scan result of point 8.

**Figure 9:**
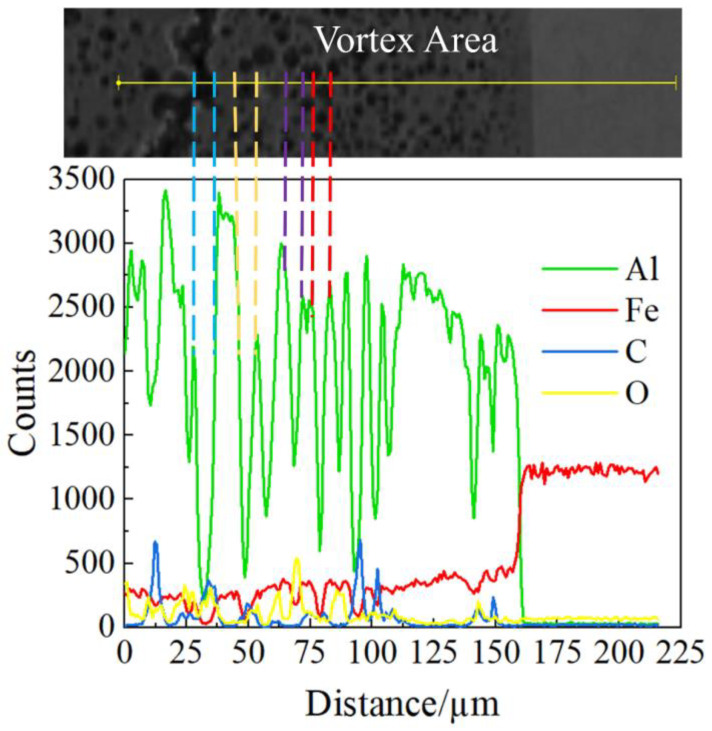
SEM scanning and EDS analysis of vortex, crack and hole.

To further analyze the distribution of elements in the unilateral vortex area of steel fiber, 8 points were selected for EDS point scanning in this area, as shown in Figure 8. The percentage of iron and aluminum is shown in Table 3. The first point and the second points were in the iron base plate, and the main components were 66.2% iron and 64.8% iron. The seventh and eighth points were in the aluminum base plate, and the main components were 88.6% aluminum, 1.3% iron, 86.9% aluminum, and 0.9% iron, respectively. There was element diffusion of the iron element in the aluminum base plate, but the diffusion of the aluminum element in the iron base plate near the interface was not obvious. The elements measured near the iron base plate were 55% aluminum, 14.4% iron, 32.3% aluminum, and 10% iron, while the elements near points 5 and 6 were 71.8% aluminum, 9.7% iron, 73.9% aluminum, and 9.3% iron. The results revealed that the elements in the vortex region were mainly aluminum and iron, and the closer to the middle region of the vortex, the higher the iron content. The main reason is that under the circumstance of high temperature and high pressure, the molten iron and molten aluminum would occur in a mutual melting phenomenon to form an iron aluminum alloy, and with the gradual decrease in temperature and pressure, the proportion of iron and aluminum in the alloy gradually changed until the aluminum and iron separated. However, because the cooling rate was too fast, the iron element in the middle of the molten region had been solidified before it was separated, so the content of iron element near the bonding interface was less than that in the middle of the vortex, which also indirectly confirmed the research results obtained by Zeng [35] and Zhang et al. [36]: intermetallic compounds were mainly formed in the vortex region on the base plate side and rarely formed on other sides of the interface. Wu Tong et al. [37,38] found that a large number of brittle intermetallic compounds produced during explosive welding were the main causes of cracking at the bonding interface. These intermetallic compounds formed cracks, pores, and voids in the vortex region, resulting in a decrease in the mechanical properties of metal composites [39,40,41]. To explore the types of metal compounds in the vortex region, EDS scanning was performed on cracks, holes, and air pores in the vortex region, and the results are shown in Figure 9. The four groups of dotted lines in Figure 9 are the line scanning results corresponding to the SEM images of pores and air holes in the vortex region. By comparing the SEM and EDS results in the figure, it was found that the content of iron and aluminum in the EDS scanning results corresponding to cracks, holes, and air pores decreased at the same time, while other elements increased. Therefore, the metal compound formed in the vortex region might be FeAl.

**Figure 10:**
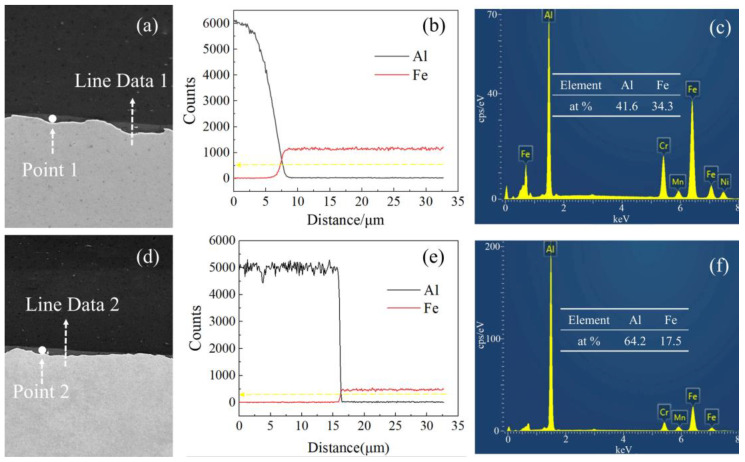
SEM and EDS results of the bonding interface of steel-aluminum composite plate: (**a**) no bonding interface of steel fiber; (**b**) figure a line scan results; (**c**) figure a point scan results; (**d**) bonding interface with steel fiber diameter of 0.5 mm; (**e**) figure d line scan results; (**f**) figure d point scan results.

To further explore the impact of the addition of steel fibers on the interface of the steel-aluminum composite plate prepared by explosive welding, EDS scanning was carried out on the interface of the steel-aluminum composite plate without steel fibers and the steel-aluminum composite plate with a steel fiber diameter of 0.5 mm, and the results are shown in Figure 10a,d, respectively, forming an obvious contrast. There was a gradual element change in Figure 10a, transiting from iron element to aluminum element. The mixing amount of elements in the transition zone reached about 500, and the content of aluminum and iron elements was 41.6% and 34.3%, respectively, as shown in Figure 10c. In Figure 10d, the transition from iron to aluminum was very fast. The mixing amount of elements in the transition zone was about 300, and the aluminum and iron contents were 64.2% and 17.5%, respectively, as shown in Figure 10f. The element transition in the group without steel fibers was more obvious and the mixing amount of the two elements in the transition zone was larger than in the steel fiber group. Studies have shown that the formation of the transition zone of chemical elements was caused by the strong stirring of jet particles, and the diffusion rate increased significantly with the increase in the defect density caused by plastic deformation [42,43,44]. Therefore, the simulation results and experimental results also simultaneously verified that the addition of steel fiber blocked the continuous action of jet particles on the base plate surface and affected the element transition at the bonding interface of the composite plate.

### 3.3. Microhardness Analysis

**Figure 11:**
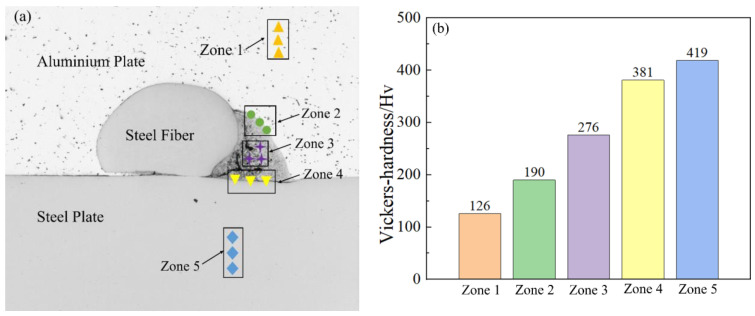
Microhardness at the interface of steel fiber-reinforced steel-aluminum composite plate: (**a**) microhardness test area; (**b**) microhardness test value.

To explore the micromechanical properties of the steel fiber-reinforced steel-aluminum composite plate, a microhardness test of the cross-section of the steel-aluminum composite plate with a steel fiber diameter of 0.5 mm was carried out. The corresponding test area was divided into five parts, as shown in Figure 11a. Among them, Zone 1 is the microhardness test area of the cross-section of the 2A12 aluminum, Zone 2 is the microhardness test area of the interface between the vortex region and 2A12 aluminum, Zone 3 is the microhardness test area of the vortex region, Zone 4 is the microhardness test area of the interface between vortex region and SS-304 stainless steel, and Zone 5 is the microhardness test area of SS-304 stainless steel. Figure 11b shows the numerical results of the microhardness values of the five zones in Figure 11a. The highest hardness was 419 Hv for the SS-304 stainless steel base plate, the lowest hardness was 126 Hv for 2A12 aluminum, the interface between the vortex region and 2A12 aluminum was 190 Hv, the interface between the vortex region and SS-304 stainless steel was 381 Hv, and the vortex region was 276 Hv. According to previous studies, the highest hardness of the composite plate prepared by explosive welding should be in the intermetallic compound layer [45,46], that is, the vortex region. However, the experimental data obtained in this paper deviated from the previous conclusions, and there might be two reasons for the decrease in the hardness in the vortex region. First of all, during the experimental process of explosive welding, a large number of high-temperature molten jet particles were strongly stirred and mixed on one side of the steel fiber to form the intermetallic compound FeAl. As the time was not enough to reduce the large-scale heat, these molten particles could not rapidly solidify, and the continuous stirring and mixing led to a large number of cracks, holes, and air pores in the vortex region, as shown in Figure 8, so the hardness of the vortex region decreased. Secondly, it can be seen from the EDS point scanning results of points 3 and 7 in Figure 8 that the interface between the vortex zone and the SS-304 stainless steel was composed of 14.4% SS-304 stainless steel and 55% aluminum, and the interface between the vortex zone and the 2A12 aluminum was composed of 1.3% SS-304 stainless steel and 88.6% 2A12 aluminum. According to the point scanning results of points 5 and 6, it can be seen that the vortex region was mainly composed of about 32% 2A12 aluminum and about 10% SS-304 stainless steel, while the microhardness of 2A12 aluminum was 126 Hv, so the microhardness of the vortex region decreased. The microhardness of SS-304 stainless steel in this test reached 419 Hv. The main reason for the significant increase in the microhardness of SS-304 stainless steel was that austenitic steel is one of the most obvious metal materials subjected to explosive strengthening. Therefore, explosive strengthening was more widely used in the strengthening process of austenitic steel than mechanical forging.

### 3.4. Mechanical Property Testing

**Figure 12:**
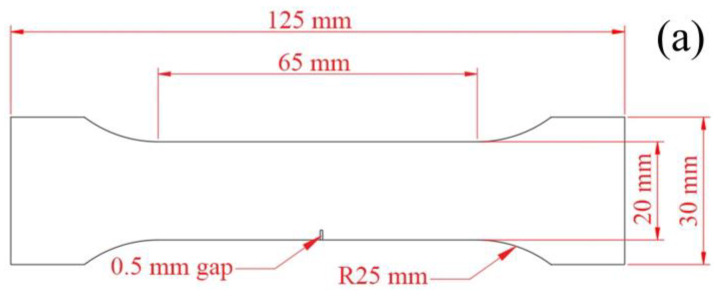

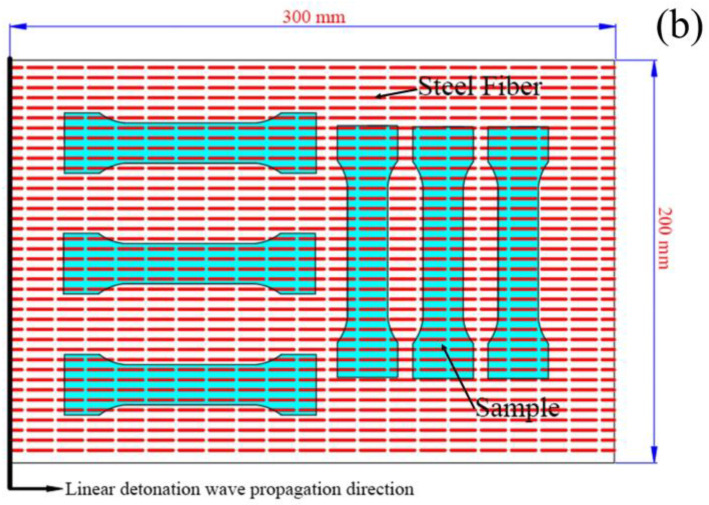
Tensile specimen parameters: (**a**) dimensional drawing of tensile specimen; (**b**) composite plate size, sampling position and linear detonation wave propagation direction diagram.

A 0.5 mm notch was opened on one side of the sample to measure the tensile strength of the sample, then the impact of the addition of steel fibers on the tensile properties of steel-aluminum composite plates prepared by explosive welding was explored. Three specimens were taken in the steel fiber parallel to the tensile load direction and the steel fiber perpendicular to the tensile load direction. The specific location and size parameters of the sample are shown in Figure 12. Then, the tensile test of the specimen was performed at room temperature by the UTM5000 series microcomputer-controlled electronic multipurpose testing machine at a loading rate of 2 mm/min. The accuracy of this series of equipment is class 0.5, and the error of test force indication of load parameters is within ± 0.5% of the indicated value. Finally, the tensile strength of the specimen was calculated by the following formula [47].

(7) $$\sigma =\frac{F}{S}$$

where *σ* is the tensile strength, *F* is the loading load, and *S* is the stressed area of the sample.

**Figure 13:**
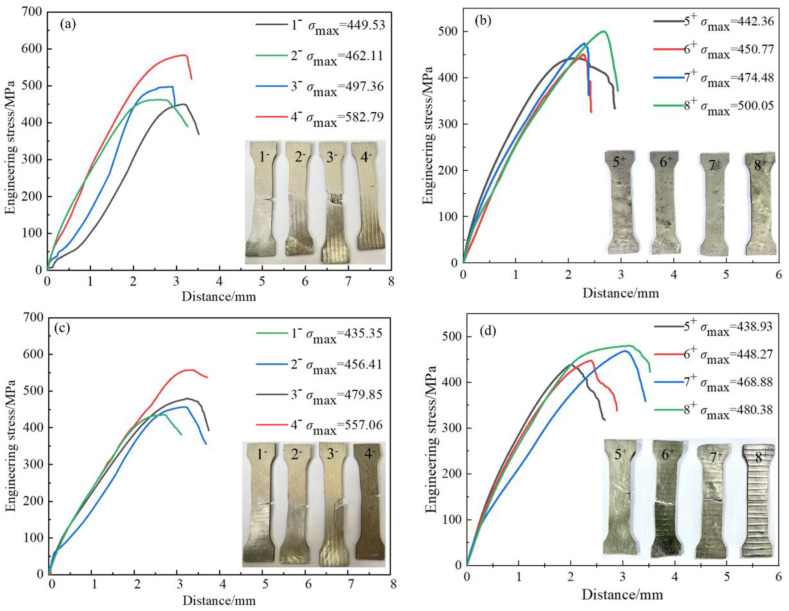

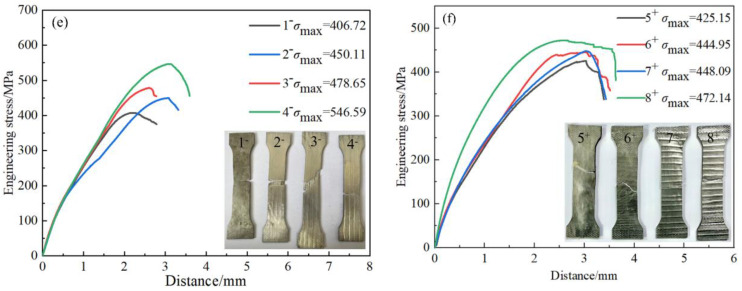
Tensile test and results: (**a**) the first group of tensile test samples and tensile data parallel to the fiber direction; (**b**) the first group of tensile test samples and tensile data orthogonal to the fiber direction; (**c**) the second group of tensile test samples and tensile data parallel to the fiber direction; (**d**) the second group of tensile test samples and tensile data orthogonal to the fiber direction; (**e**) the third group of tensile test samples and tensile data parallel to the fiber direction; (**f**) the third group of tensile test samples and tensile data orthogonal to the fiber direction.

The samples in each group were from the same position in the center of four steel-aluminum composite plates prepared by explosive welding, aiming to avoid the influence of boundary effect on tensile samples. In Figure 13 there were no steel fibers in 1^−^ and 5^+^ samples, and the fiber diameters in 2^−^ and 6^+^, 3^−^ and 7^+^, 4^−^ and 8^+^ samples were 0.25 mm, 0.35 mm, and 0.5 mm, respectively. To ensure the accuracy of the experimental data, each sample of the steel fiber parallel to the tensile load direction contained four steel fibers.

Table 4 shows the specific parameters of the tensile resistance of the steel fiber-reinforced steel-aluminum composite plate. “−” and “+” indicate that the steel fiber is parallel and perpendicular to the tensile load direction, respectively. In the direction of the steel fiber parallel to the tensile load, the average tensile strength of sample 1^−^ was 420.53 MPa. The average tensile strength of sample 2^−^ increased by 8.5% compared with sample 1^−^, with the highest increase of 9.8% and the lowest increase of 7.0%; the average tensile strength of sample 3^−^ increased by 15.4% compared with sample 1^−^, with the highest increase of 18.3% and the lowest increase of 13.8%. The average tensile strength of sample 4^−^ increased by 33.7% compared with sample 1^−^, with the highest increase of 38.6% and the lowest increase of 29.9%. In the direction of the steel fiber perpendicular to the tensile load, the average tensile resistance of sample 5^+^ was 435.48 MPa, and the average tensile resistance strength of sample 6^+^ was 2.9% higher than that of sample 5^+^, with the highest increase of 3.5% and the lowest increase of 2.2%. Compared with sample 5^+^, the average tensile resistance strength of sample 7^+^ increased by 6.5%, with the highest increase of 8.9% and the lowest increase of 2.2%. Compared with sample 5^+^, the average tensile resistance strength of sample 8^+^ increased by 11.2%, with the highest increase of 14.8% and the lowest increase of 8.4%. The main difference between samples 1^−^, 2^−^, 3^−^, 4^−^ and samples 5^+^, 6^+^, 7^+^, 8^+^ was that the first four groups of samples were parallel to the direction of the tensile load and along the direction of the detonation wave propagation, while the last four groups were perpendicular to the direction of the tensile load and the detonation wave propagation. Among them, the sample groups 1^−^ and 5^+^ were from the steel-aluminum composite plates without steel fibers. When the highest and lowest tensile resistance properties were removed from the two groups of data, they were 435.35 MPa and 438.93 MPa, respectively. The difference in the tensile resistance between the two groups could be neglected, so the influence of the propagation direction of the detonation wave on the tensile resistance was excluded. In summary, the addition of steel fibers could improve the tensile resistance of steel-aluminum composite plates prepared by explosive welding. With the increase in steel fiber diameter, the tensile resistance strength increased gradually. Agraw Ryuichi [15] used explosive welding to produce a fiber composite consisting of a high-strength plastic steel wire and a pure aluminum or titanium matrix, and then verified the strength of the composite by tensile tests. The results revealed that the optimum tensile resistance load was determined by the volume of the fiber part, that is, the larger the volume of fibers added, the stronger the tensile resistance. Taking AMr6 aluminum alloy as the base plate and unidirectional fiber composited by steel wire as the reinforced composite material, Kotov V A [15] pointed out that the strength of this material depended on the internal geometric installation under uniaxial and biaxial loads in many cases. When the load was parallel to the fiber, the fiber had a particularly large effect on the composite. The results revealed that the fiber-reinforced composites and tubular products prepared by explosive welding had high enough strength under various loads. Explosion strengthening is using the force generated by explosives to replace mechanical forging so that the strength of steel is improved to a certain extent, and the effect of austenitic steel is more obvious when subjected to explosion strengthening process. Therefore, steel fiber was selected as the steel fiber-reinforced phase. The strengthening of metal composite materials prepared by explosive welding mainly includes two aspects: (1) after explosive welding, the base plate, composite plate, and steel fiber were all subjected to explosion strengthening, so their tensile strength was improved; (2) the overall tensile resistance strength of the composite material after explosive welding was better than that of the base plate metal [48]. With the addition of steel fibers, the tensile resistance of steel-aluminum composite plates was greatly increased. The tensile resistance of the steel fiber parallel to the tensile load direction was much larger than that perpendicular to the tensile load direction. Therefore, the tensile resistance of steel fiber-reinforced steel-aluminum composite plates was greatly improved. The main reasons are as follows. (1) During the explosive welding process, the tensile resistance of the steel fiber was improved due to the explosion strengthening. (2) The addition of steel fiber improved the overall tensile resistance of steel fiber-reinforced steel-aluminum composite plates.

### 3.5. Fracture Appearance Analysis

**Figure 14:**
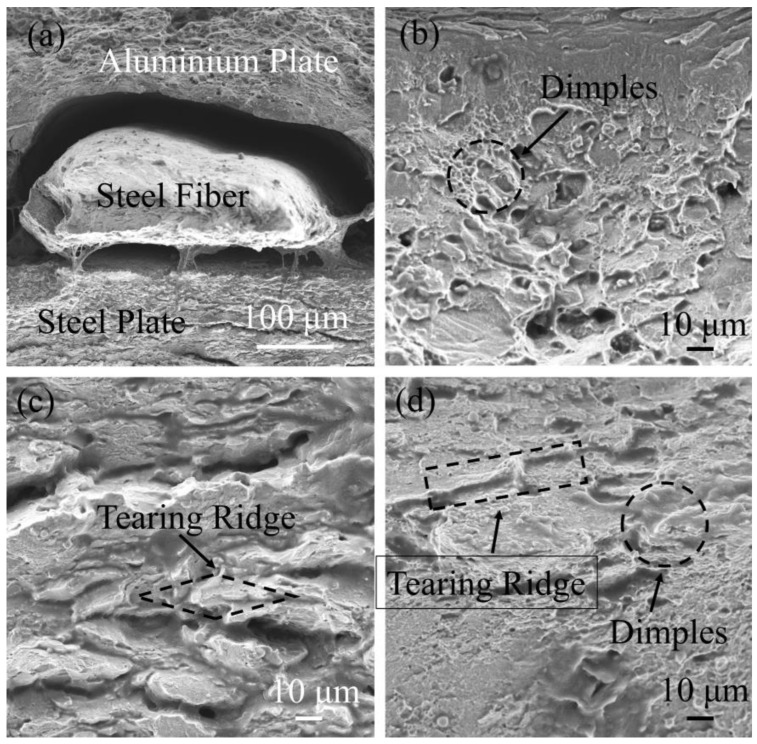
Fracture SEM of fiber-reinforced composite plate: (**a**) fracture SEM; (**b**) 2A12 aluminum fracture SEM; (**c**) SS-304 stainless steel fracture SEM; (**d**) fiber fracture SEM.

SEM was used to analyze the fracture of the tensile specimen and study the failure mechanism of steel fiber-reinforced steel-aluminum composite plates. Figure 14a shows the overall morphology of the fracture of the tensile specimen. From top to bottom, the layers are 2A12 aluminum, S20910 steel fibers and SS 304 stainless steel. The overall fracture morphology is relatively flat, but small tough nests and quasi-dissociative fracture openings can still be observed, which proves that different fractures have occurred in the different materials. Meanwhile, it can be observed that the steel fibers have separated from the aluminum layer and remain bonded to the steel layer. Figure 14b shows the fracture morphology of the 2A12 aluminum layer, from which it can be observed that the fracture surface is hole-like, the hole is shallow and the edge of the hole is parabolic, showing an obvious dimple shape. Therefore, the fracture of the 2A12 aluminum layer is a ductile fracture, so it is worth noting that near the edge of the welding line, the dimple morphology of the 2A12 aluminum layer gradually changed, and the depth of the hole gradually became shallow. The main reason is that the composite plate was subjected to explosion strengthening during the explosive welding process, and as the deformation increased, the strength of the explosive strengthening also gradually increased, which also verified that the maximum deformation in the numerical simulation results occurred at the bonding interface, as shown in Figure 6c. The microhardness at the bonding interface between the 2A12 aluminum and steel fiber was also greater than that of the 2A12 aluminum, which further confirmed that the bonding interface was subjected to explosion strengthening, as shown in Figure 11. Figure 14c shows the fracture morphology of the steel layer under high multiples. Obvious tearing edges and holes can be observed, and there is no flat cleavage section, so the steel layer has a quasi-cleavage fracture. The fracture morphology of the steel fiber is shown in Figure 14d, from which it can be seen that the fracture of steel fiber is relatively flat, and there are small dimples and obvious tearing edges on the surface, so the steel fiber also has a quasi-cleavage fracture. Meanwhile, it can also be observed that not all the steel fiber is extracted, and the local section is reduced while maintaining the connection with the base plate and the composite plate, and it was judged that the steel fiber had a necking phenomenon. This is because the steel fiber has not completely broken after the fracture behavior of the plate during the stretching process, and the necking phenomenon was finally broken by stretching.

## 4. Conclusions

The steel fiber-reinforced steel-aluminum composite plate was successfully prepared by explosive welding, and the interface evolution mechanism of the steel-aluminum composite interface after adding steel fiber was simulated by the SPH method. Then, steel fiber-reinforced steel-aluminum composite plates were further studied using SEM, EDS, and tensile tests, and the following conclusions were drawn.

(1) The SPH simulation method proved that there was a certain angle formed by the steel fiber, the base plate, and the composite plate, which blocked the direction of the jet so that the jet accumulated on one side of the steel fiber to form an environment with high temperature, high pressure, and high strain rate, thereby forming a vortex area. The residual stress of the jet particles in the vortex region forced them to undergo strong stirring, producing cracks, holes, and air pores in the vortex region.
(2) SEM and EDS analysis proved the following two points. 1. The main reason for cracks, holes, and air pores in the vortex area was the formation of a large number of brittle intermetallic compounds, and the brittle intermetallic compound was mainly FeAl. 2. due to the blocking effect of steel fiber on the jet, the transition of elements at the interface of the composite plate with steel fiber was relatively fast.
(3) Microhardness test results revealed that the cracks, holes, and air pores in the vortex region influenced the microhardness of the bonding interface to a certain extent. As the microhardness of 2A12 aluminum was relatively small, the microhardness increased with the decrease in 2A12 aluminum content in the vortex region.
(4) The tensile test on the universal specimen machine proved that adding steel fibers could improve the antitensile properties of the steel-aluminum composite plates. The larger the diameter of steel fiber, the more obvious the tensile properties are. The main reason is that the steel fiber material is S20910 stainless steel, which was significantly strengthened during the explosion process.
(5) Fracture morphology analysis indicated that 2A12 aluminum had a typical ductile fracture. The ductile fracture far from the bonding interface was more obvious because of explosion strengthening. The SS-304 stainless steel layer and steel fiber were quasi-cleavage fractures, and the steel fibers showed a necking phenomenon during fracture.

## Figures and Tables

**Table 1:** Performance parameters of ANFO.

Material	Density *ρ*/(g cm^−3^)	Detonation Velocity D/(m s^−1^)	Specific Internal Energy E0/(kg cm^−3^)	Detonation Pressure /MPa	Heat Capacity Ratio
ANFO	0.681	2430	2.484	1160	2.5

**Table 2:** Performance parameters of metal materials [24,25].

Material	Density *ρ/*(g·cm^−3^)	Yield Strength *σ*_b_/MPa	Vickers Hardness /Hv	Wave Velocity *C*_0_/(m·s^−1^)	Melting Point *T*/K	Specific Heat *C_p_* /(J·kg^−1^·K^−1^)	Thermal Conductivity *K*/(W·m^−1^·K^−1^)
2A12 aluminum	2.79	354	110	5328	933	940	237
S20910 steel	7.88	380	201	4569	1450	476	14
SS-304 steel	7.93	515	200	5790	1400	500	21.5

**Table 3:** Iron and aluminum contents at 8 EDS points.

Element	1	2	3	4	5	6	7	8
iron	66.2%	64.8%	14.4%	10%	9.7%	9.3%	1.3%	0.9%
aluminum	0	0	55%	32.2%	71.8%	73.9%	88.6%	86.9%

**Table 4:** Tensile performance parameters of steel-aluminum composite plates.

Serial No.	First Group of Data/MPa	Second Group of Data/MPa	Third Group of Data/MPa	Mean Value/MPa
1^−^	449.53	435.35	406.72	420.53
2^−^	462.11	456.41	450.11	456.21
3^−^	497.36	479.85	478.65	485.29
4^−^	582.79	557.06	546.59	562.15
5^+^	442.36	438.93	425.15	435.48
6^+^	450.77	448.27	444.95	447.99
7^+^	474.48	468.88	448.09	463.80
8^+^	500.05	480.38	472.14	484.19

## Data Availability

Data are contained within this article.
